# Layered Double Hydroxide Nanoparticles to Overcome the Hydrophobicity of Ellagic Acid: An Antioxidant Hybrid Material

**DOI:** 10.3390/antiox9020153

**Published:** 2020-02-13

**Authors:** Szabolcs Muráth, Adél Szerlauth, Dániel Sebők, István Szilágyi

**Affiliations:** 1 MTA-SZTE Lendület Biocolloids Research Group, University of Szeged, H-6720 Szeged, Hungary; murathsz@chem.u-szeged.hu (S.M.); szerlauth.adel@gmail.com (A.S.); 2 Department of Physical Chemistry and Materials Science, Interdisciplinary Excellence Center, University of Szeged, H-6720 Szeged, Hungary; 3 Department of Applied and Environmental Chemistry, Interdisciplinary Excellence Center, University of Szeged, H-6720 Szeged, Hungary; sebokd@chem.u-szeged.hu

**Keywords:** 2D antioxidant biomaterial, ellagic acid, layered double hydroxide, immobilization, radical scavenging

## Abstract

Ellagic acid (EA), a polyphenolic antioxidant of poor water solubility, was intercalated into biocompatible layered double hydroxide (LDH) nanoparticles by the coprecipitation method. Structural investigation of the composite revealed that the lactone bonds split under the synthetic experimental conditions, and EA was transformed to 4,4′,5,5′,6,6′-hexahydroxydiphenic acid during intercalation. To improve the surface properties of the EA-LDH composite, the samples were treated with different organic solvents. The antioxidant activity of the LDH hybrids was assessed in test reactions. Most of the obtained hybrids showed antioxidant activity comparable to the one of the free EA indicating that the spontaneous structural transformation upon immobilization did not change the efficiency in radical scavenging. Treatments with organic solvents influenced the activities of the materials remarkably. The main advantage of the immobilization procedure is that the products can be applied in aqueous samples in high concentrations overcoming the problem related to the low solubility of EA in water. The developed composites of high antioxidant content can be applied as efficient reactive oxygen species scavenging materials during biomedical treatments or industrial manufacturing processes.

## 1. Introduction

Layered double hydroxides (LDH) represent the only inorganic layered materials with anion-exchange capacity found in nature. Their lamellae typically consist of divalent and trivalent metal ions, theoretically derived from isomorphous replacement of Mg(II) with Al(III) ions in the structure of layered Mg(OH)_2_, although a wide range of metallic compositions may be achieved [1]. The incorporation of cations of higher charge (Al(III), for instance) leads to the formation of positively charged layers. The general formula is stated as [M(II)_1–x_M(III)_x_(OH)_2_][A^n–^·mH_2_O], where M(II) and M(III) are divalent and trivalent metal ions and A^n–^·mH_2_O is the interlamellar (i.e., intercalated), charge-neutralizing anion in hydrated state [2]. Through tuning the composition of LDH and their composites with other materials [3], they can be used in a variety of applications including adsorbents for water purification [4,5], light emitters [6], corrosion-resistant coating material [7], contrast agents [8], solid support for bioactive substances [9,10], catalyst carriers [11] and catalysts in organic reactions [12,13], or in water splitting [14,15].

Intercalation of larger anions, such as biomolecules of various activities, gives rise to the development of hybrid materials with potential applications in biomedical processes. There are established ways to produce pillared LDH with non-steroidal drugs, such as diclofenac or ketoprofen [16] and indometacin or flurbiprofen [17], while the controlled release of the immobilized anticancer agent methotrexate was also demonstrated [18]. More profound effects were achieved with combined intercalation of anticancer drugs, e.g., fluorouracil and dactolisib [19]. Folic acid was incorporated in the structure of doxorubicin-modified LDH, and the presence of this targeting molecule improved the uptake in cancer cells, as shown in in vitro tests [20]. Furthermore, carborane-containing hybrids have potential applications in boron neutron capture therapy of cancer [21].

Antioxidant materials attract widespread contemporary interest due to the growing demand in biomedical as well as industrial processes, where reactive oxygen and nitrogen species cause significant damage [22,23,24,25,26,27]. Molecular (or non-enzymatic) anionic antioxidants, such as polyphenols and carboxylic acid derivatives, may also be capsulated in LDH. Electrostatic interactions lead to stable composites. Thus, the intercalated organic materials, e.g., carnosine, gallic acid [20], and 3-(3,5-di-tert-butyl-4-hydroxy-phenyl)-propionic acid [28] maintain their radical scavenging activity. Among polyphenols, ellagic acid (EA) has generated notable research interest due to its high antioxidant activity and potential human applications, e.g., treatment of liver diseases [29]. EA commonly occurs in nature and shares from 2–10% (blueberry and currant) up to 75–90% (cloudberry and raspberry) of total polyphenol amount in various edible fruits [30]. Its content peaks in cloudberry with 0.2% of total mass and is also relevant in the antioxidant property of pomegranate [31].

It was demonstrated that EA plays a role in the chemoprevention of rat colon carcinogenesis, especially in combination with other anticancer drugs [32]. In addition, a synergistic effect between EA and quercetin was shown to induce apoptosis in human leukemia cells [33]. Recently, its protective feature was demonstrated against acrylamide, a compound with dangerous exposure symptoms related to peripheral neurotoxicity [34].

Despite the numerous positive effects, the main drawback of EA applications is the poor water solubility [35], which has also been an issue in soda pulping of eucalyptus, where greenish deposits (EA and its salts with divalent cations) clog the pipes [36]. Taken orally, the precipitation in gastric juice and relatively fast disposition of EA was shown, and the better uptake of its more soluble derivatives has been reported [37]. The bioavailability of EA was improved by encapsulation, successfully using zinc hydroxide carrier [38], collagen-chitosan matrix [39], and hydrophilic dendrimers [40] to overcompensate the solubility barrier. However, no comprehensive studies have been reported yet, where it was unambiguously confirmed that the antioxidant activity of EA-nanoparticle composites can be preserved. Using such bioactive hybrid materials, high antioxidant content can be provided in aqueous samples, e.g., in medical treatments or formulation procedures in the food and cosmetic industry.

In this research, for the first time, Mg_2_Al-LDH (denoted simply as LDH in the text), an inexpensive and biocompatible host [41], was used to mask the highly lipophilic property of EA. The structure of the novel EA-LDH composite material was also modified with organic solvents after synthesis, and the antioxidant activity of the different products was compared in two assays.

## 2. Materials and Methods

### 2.1. Materials

Magnesium(II) nitrate hexahydrate (Mg(NO_3_)_2_·6H_2_O), aluminum(III) nitrate nonahydrate (Al(NO_3_)_3_·9H_2_O), ellagic acid (EA), 4 M sodium hydroxide solution (NaOH), 1 M hydrochloric acid (HCl), ammonium acetate (NH_4_OAc), 2,2-diphenyl-1-picrylhydrazyl radical (DPPH), copper(II) chloride dihydrate (CuCl_2_·2H_2_O), neocuproine (Nc), Trolox (6-hydroxy-2,5,7,8-tetramethylchroman-2-carboxylic acid), methanol (MeOH), ethanol (EtOH), acetone (AC), acetonitrile (ACN), formamide (FA), *N*,*N*-dimethylformamide (DMF) and dimethyl sulfoxide (DMSO) were all purchased from VWR International (Radnor, Pennsylvania, USA) in analytical purity and were used as received. Purified water was produced by reverse osmosis and UV irradiation (VWR Puranity TU 3+ UV/UF system from VWR International).

### 2.2. Preparation of Hydrotalcite Intercalated with EA

**Figure 1:**
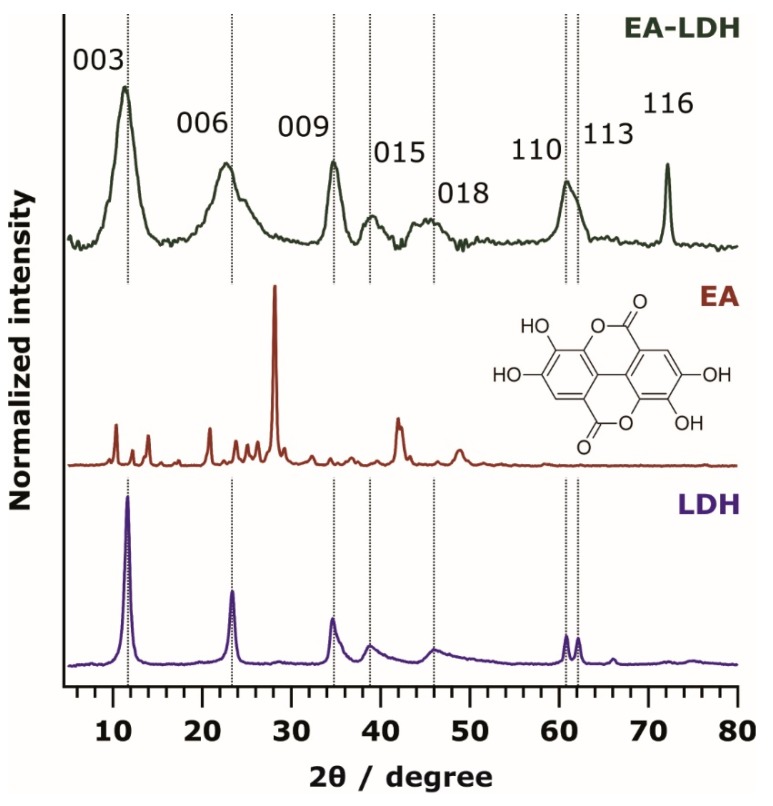
Powder XRD pattern of ellagic acid-layered double hydroxide (EA-LDH), EA, and LDH. The chemical structure of EA is shown in the inset. The Miller indices are indicated.

The intercalation of the organic material was accomplished by coprecipitation [2]. A mixed metal nitrate solution was prepared by dissolving the salts in water with c(Mg(II)) = 0.2 M and c(Al(III)) = 0.1 M. This solution was thereafter added to the mixture of EA (625 μmol, structure is displayed in Figure 1) with 4 M NaOH. The EA-to-Al(III) molar ratio was 0.25, and a final pH of 13 was maintained during the reaction. Under these conditions, complete intercalation of the added EA was observed. The core structure of EA remains the same in the presence of a strong base. However, double deprotonation at pH above 12 and lactone ring opening may occur [42]. The slurry obtained was vigorously stirred at room temperature for 24 h, and the precipitate was separated by centrifugation at 4200 rpm (2090 rcf) for 10 min using an Orto Alresa (Madrid, Spain) Unicen 21 device. The solid material was washed three times with water and then dried at 50 °C overnight. Hydrothermal treatment (HT) was also applied for the EA-LDH obtained. During this method, stirring was stopped after 1 h, and the slurry was transferred to an autoclave with Teflon lining (Col-Int Tech, Irmo, S.C., USA) and was treated at 120 °C for 24 h in an oven. After cooling to ambient temperature, the sample was separated and purified as detailed earlier. Reference LDH was prepared using the same method, but without introducing EA or HT.

### 2.3. Aqueous Miscible Organic Solvent Treatment (AMOS-T) of the EA-LDH

Six solvents (MeOH, EtOH, AC, ACN, FA, and DMF) were applied to modify the surface properties of the organic-modified LDH [43]. In a typical method, the EA-LDH was synthesized as detailed before, and the aqueous washing was followed by a two-step stirring in 25–25 mL of the respective solvent for 2 h and 1 h each. Between the disjoint steps, the slurry was separated by centrifugation (4200 rpm, 2090 rcf, 20 min), and a fresh solvent aliquot was used. Finally, the modified EA-LDH composites were obtained after centrifugation (4200 rpm, 2090 rcf, 20 min) and drying at 50 °C overnight. Overall, 9 materials were prepared, 8 of which contained EA (Table 1).

### 2.4. Instrumental Characterization of the LDH

The X-ray diffractometry (XRD) measurements of the finely ground samples were performed on a Bruker (Billerica, MA, USA) D8 Advanced diffractometer with CuK_α_ (λ = 0.1542 nm) as the radiation source at ambient temperature in the 5–80° (2θ, where θ is the incidence angle of the beam) range applying 0.02° step size. The basal spacing of the LDH was calculated by the Bragg’s relation (Equation S1).

Morphological analyses were conducted using a Hitachi (Tokyo, Japan) S-4700 scanning electron microscope (SEM) at various magnifications with 10 kV accelerating voltage after gold deposition on the surface.

The Fourier transformed infrared (FT-IR) spectra of the solids were acquired using a JASCO (Easton, MD, USA) FTIR-4700 spectrometer with a DTGS (deuterated triglycine sulfate) detector in attenuated total reflectance (ATR, ZnSe accessory) mode. The spectral resolution was 1 cm^−1^, and a total number of 128 scans were collected for a spectrum. The noise of carbon-dioxide was removed by the built-in software package, and the spectra were baseline-corrected and smoothed.

UV-visible concentration measurements were conducted on a Thermo Fischer Genesys (Waltham, MA, USA) 10S dual beam spectrophotometer, equipped with a xenon flash lamp and a silicon photodiode detector.

The specific surface area of the LDH samples was determined using the BET (Brunauer–Emmett–Teller) method by measuring N_2_ adsorption isotherms at 77 ± 0.5 K with a Micromeritics Gemini (Norcross, GA, USA) 2375 Surface Area Analyzer instrument. Before measurements, the samples were evacuated at 10^−5^ mm Hg and 100 °C overnight.

Dynamic light scattering measurements were performed to measure the size of the LDH and the hybrid particles in dispersions. The experiments were performed on a LiteSizer 500 device (Anton Paar, Graz, Austria) at a 175° scattering angle, applying the second cumulant fit to the correlation function. The hydrodynamic radii were calculated using the Stokes–Einstein equation [44].

### 2.5. Determining the EA content of the EA-LDH

The visible absorbance of EA at λ = 350 nm was measured after reacting a portion of the LDH obtained with 1 M HCl. Upon complete dissolution, the sample was diluted with a 50 V% aqueous MeOH solution, and the measurements were carried out at pH = 2.5. The calibration curve was acquired using a 30 mg/L EA stock solution in 50 V% MeOH solution at the same pH. The effect of the protonation state of EA to its UV-Vis spectrum was investigated by setting a pH of about 13 and 2 in a stirred cuvette. At these pH values, EA is present in an opened, deprotonated form (above pH 12) and in a fully protonated, lactone form (below pH 3). For the concentration measurements, 1 M NaOH (for basic pH) and 1 M HCl (for acidic pH) was added to a methanolic solution of EA to screen the UV-Vis spectra at different pH values.

### 2.6. Antioxidant Activity of the Hybrid Materials

The antioxidant property of the EA-LDH was monitored in the DPPH (2,2-diphenyl-1-picrylhydrazyl free radical) test reaction and the CuPRAC (cupric reducing antioxidant capacity) assay. The former one relies on the scavenging of commercially available DPPH free radicals [45], while the latter is based on the reducing potential of the material in question against neocuproine-chelated Cu(II) ions [46].

For the DPPH test, 3500 μL of 60 μM DPPH solution (in methanol, freshly prepared daily) was completed to 3600 μL with water and with the aqueous suspension (5 g/L) of the LDH. The reference measurement was executed with EA dissolved in DMSO (c = 4.32 mM) due to the poor water solubility. The absorbance decrease was followed at λ = 517 nm, the absorption maximum of DPPH, at various EA-to-DPPH molar ratios. The measurement time was 45 min for EA and 60 min for the EA-LDH hybrids. The Beer–Lambert equation was used for quantifying the remainder of DPPH from the initial absorbance (Equation S2).

For the CuPRAC method, 500 μL solution of CuCl_2_ (10 mM), Nc (7.5 mM in ethanol), and NH_4_OAc buffer (pH = 7, 1 M) were mixed in a cuvette. The reaction mixture was completed with 550 μL, 1 mM aqueous EA-LDH suspensions, and with water. For reference, the DMSO solution of EA was used in 1 mM concentration as well. The cuvettes were set aside for 30 min (until the reaction finished), and the absorbance was measured at λ = 450 nm, the absorption maximum of the Cu(I)Nc_2_ complex formed.

For both methods, the absorbance values recorded at each point were corrected by the absorbance of the dispersed LDH, as they had a slight contribution due to light scattering phenomena. Measurement errors were assessed for both methods by triple repetition at 5 different concentrations, using EA-LDH. For data evaluation, Microsoft (Redmond, WA, USA) Excel 365 software was used to fit exponential (DPPH assay) and linear correlations (CuPRAC assay).

### 2.7. Release of EA from the Composites

The possible leakage of the intercalated substance was followed via UV-Vis spectrophotometry. The LDH hybrids were vigorously stirred in the simulated medium of the antioxidant assays (50 V% MeOH in water) in 5 g/L concentration for 60 min. At five different time intervals, 6 mL aliquots were sampled and filtered by a syringe filter (100 nm pore size) to remove the floating solid particles. Thereafter, the pH of the solution was set with 1 M HCl, and the EA content was measured as detailed in Section 2.5.

## 3. Results and Discussion

### 3.1. Structural Features

To prove the successful synthesis of the LDH and EA-LDH materials, XRD measurements were performed. The distinctive XRD pattern of the pristine LDH is shown in Figure 1 with the pattern of EA and EA-LDH.

The diffractogram of EA-LDH indicates that only 1 phase material was formed, and it contained the characteristic peaks reported earlier for LDH-based material [2]. It is obvious from the results that co-crystallization of EA and LDH did not occur during the synthesis, as only the peaks of LDH are present. Higher peak widths of the EA-LDH in comparison to the reference LDH indicate the formation of materials with somewhat poorer crystallinity. The intercalation of larger anions often leads to significantly increased layer distances [47,48,49,50], which may be reflected by the shift of the (003) peak towards lower 2θ values. A similar shift was present only to a small extent in the EA-LDH diffractogram. Therefore, it is likely that the guest EA ion is situated in a planar configuration between the layers without forming a pillared LDH. Considering the usual interlayer distance reported for Mg_2_Al-LDH materials [51], a maximum space of 2.8 Å remains for the intercalants, which is large enough for anions with planar or nearly planar shape, such as EA in the present case. Such a flat conformation of the intercalated substances was also recommended earlier [52,53,54]. Such a conformation is expected due to its high-degree of conjugation. Apart from intercalation, EA adsorption on the outer LDH surface may have occurred during the syntheses.

As shown in Figure S1 and Table S1, the main structural features remained very similar in the XRD patterns after AMOS treatments (aqueous miscible organic solvent treatment, AMOS-T) of the EA-LDH. A basal spacing close to 7.5–7.9 Å was registered for all LDH, indicating slight increases after intercalation. These data shed light on the fact that washing the EA-LDH with AMOS did not significantly affect the lamellar structure of the materials.

**Figure 2:**
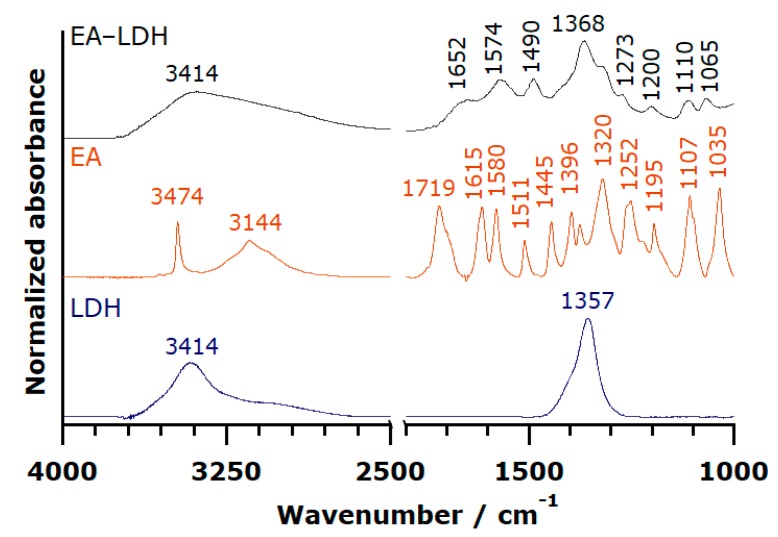
FT-IR spectra of EA-LD, EA, and LDH.

The presence of organic matter in the LDH was proved by FT-IR spectroscopy by detecting and assigning the characteristic vibration bands of the individual substances in the composites. The spectra of the LDH support, EA, and EA-LDH hybrid material are shown in Figure 2.

**Figure 3:**
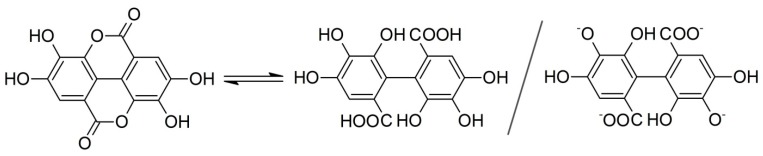
Chemical equilibrium between EA and its opened form, 4,4′,5,5′,6,6′-hexahydroxydiphenic acid, and its potential intercalated form.

While the spectrum of pure LDH and EA differed, the detail-rich region of EA from 1800 to 1000 cm^–1^ was represented on the spectrum of EA-LDH confirming the presence of EA in the composite. The sharp band of C=O stretching (typical for esters) vibration at 1719 cm^–1^ disappeared during the intercalation reaction in highly alkaline solution, implying spontaneous ring openings at the lactone bonds. Therefore, the intercalated ion is probably the deprotonated form of the opened EA, 4,4′,5,5′,6,6′-hexahydroxydiphenic acid (Figure 3) with close to planar geometry. Since this opened form exists only in highly alkaline samples, the data were only comparable with the one obtained for EA.

The whole set of spectra, including the ones after AMOS treatments, are presented in Figure S2, and the full assignment of the FT-IR bands is tabulated in Table S2. The IR spectra of AMOS-T EA-LDH were identical to the original EA-LDH, indicating that the structure of the intercalated molecules did not change significantly upon washing with organic substances. Note that the incorporation of the solvent molecules is possible into the composites materials. However, the bands of the intercalated or adsorbed EA were indistinguishable from the ones corresponding to the AMOS. The above results, together with the XRD studies, clearly show that the AMOS-T changed neither the lamellar structure of the host nor the nature of the guest molecule. Signals for the presence of carbonate traces could be observed in the spectra. However, these molecules did not affect the EA intercalation and adsorption processes significantly.

**Figure 4:**
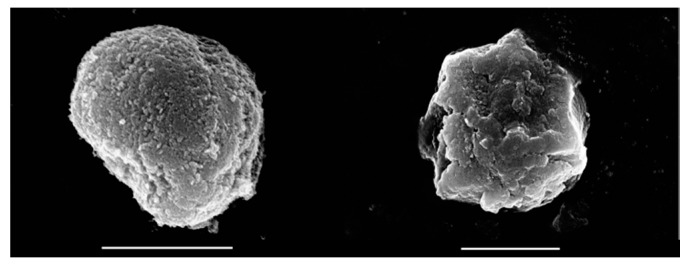
SEM micrograph of LDH (left) and EA-LDH (right) at 25,000× and 18,000× magnification, respectively. Scale bars represent 2 μm.

To probe the possible effect of the washing steps on the morphology of the obtained hybrid materials, SEM micrographs were recorded. In Figure 4, the images of the LDH and EA-LDH are depicted, and the other modified LDH is shown in Figure S3.

Based on the SEM experiments, one can conclude that the synthesized materials present similar features irrespective of the intercalation process or the AMOS treatments. In each case, the samples consisted of similar aggregates in solid-state, built up from smaller, irregulate grains. These morphological properties are common amongst LDH containing Mg(II) and Al(III) metal ions in the layers [55,56].

To determine the size of the individual LDH particles and of the EA intercalated derivatives after AMOS treatments, dynamic light scattering experiments were performed in aqueous dispersions. The obtained data (Table S1) indicated that the hydrodynamic radius of the as-prepared LDH was about 96 nm, while the radii of the EA containing samples were between 282 nm and 559 nm. These primary particles build up the aggregates observed by SEM in solid-state.

### 3.2. Determination of Antioxidant Content

After the acidic dissolution of the composites and appropriate dilution, the EA content of the LDH was evaluated by quantitative absorbance measurements. First, the reversibility of the lactone ring opening and closure was explored. Although the IR spectrum of EA undergoes changes that indicated the hydrolysis of the lactone bond, it reformed within seconds after the addition of HCl. This was proved by UV-Vis measurements, as the spectra of the two forms differed (Figure S4). Due to the dynamic equilibrium, the antioxidant content of EA-LDH could be measured in an acidic medium.

Using the appropriately recorded calibration curve of EA (Figure S5), the antioxidant contents of the samples were calculated, and most of them fluctuated in a narrow window between 16.4 and 17.2 mass% (Table S3). However, the HT-EA-LDH and FA-EA-LDH contained relatively higher (18.9 mass%) and lower (14.7 mass%) amounts of immobilized EA, respectively. For HT-EA-LDH, the source of the higher EA content is attributed to the more effective adsorption at elevated temperatures. On the other hand, formamide is known as a potential delaminating agent of LDH [57,58,59], and thus, a temporary structural disruption might lead to lower EA loading.

The overall high EA content clearly indicates that intercalation was more prominent than adsorption on the outer surface. This fact is further confirmed by the comparison of the EA and Al(III) content of the LDH since one can deduce that approximately 50% of the anion exchanging positions were occupied by EA or 4,4′,5,5′,6,6′-hexahydroxydiphenic acid after the ring opening reaction (Figure 3). This fraction would be significantly lower if EA adsorbs solely on the outer surface. The estimate that 50% of anion exchange positions were occupied by the organic anions is the result of the quantity of Al(III) ions (responsible for anion exchange) and the intercalated species, assumed to form anions with four negative charges.

### 3.3. Antioxidant Activity of EA-LDH and Leakage of EA

**Figure 5:**
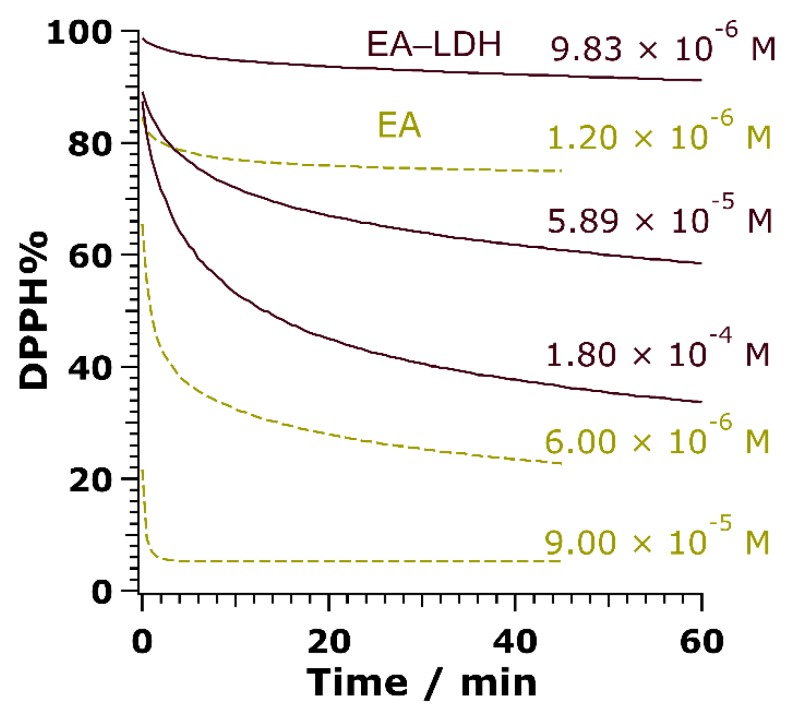
Changes in DPPH concentration in the presence of EA (dashed lines) and EA-LDH (full lines) as a function of the reaction time at 3 different initial antioxidant doses.

To assess the radical scavenging activity of the composites, the redox reaction between the antioxidant compounds and DPPH radicals was tested (Figure S6). First, the reaction rate between bare or immobilized EA and DPPH had to be uncovered. The unmodified LDH showed no activity (not shown). Accordingly, purple colored DPPH reacts with the –OH groups of EA to transform into a light-yellow solution of DPPH-H, which does not contain radical. While bare EA in DMSO neutralized the free radicals in a moderate to fast manner depending on the concentration applied, the reaction reached a steady-state with a moderate rate using EA-LDH as aqueous colloids (Figure 5). The time difference until reaction completion for the free and the immobilized EA probably emerged from the poorer availability of EA anions between the lamellae.

**Figure 6:**
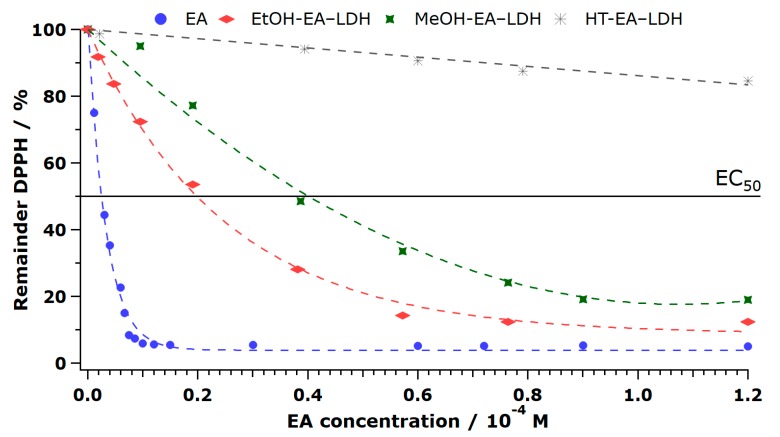
Percentage of remaining (non-reacted) 2,2-diphenyl-1-picrylhydrazyl radical (DPPH) at steady-state as a function of the EA concentration applied. EC_50_ indicates the EA concentration necessary to decompose 50% of DPPH. Measurements have an error of about 3%.

Plotting the remainder of DPPH (Equation S2) at steady-state versus the concentration of EA at the start of the reaction (calculated from the EA content of LDH, as described earlier), the antioxidant materials behaved differently, and the activities depended strongly on the solvents used in the AMOS treatments. Accordingly, EA, ACN-EA-LDH, and EtOH-EA-LDH showed high activity, AC-EA-LDH, DMF-EA-LDH, and MeOH-EA-LDH showed mediocre activity, while low activity was assigned to EA-LDH. In addition, HT-EA-LDH and FA-EA-LDH showed very low ability in scavenging DPPH radicals (Figure 6 and Figure S7). The very low scavenging of HT-EA-LDH and FA-EA-LDH means that they did not decompose half of the DPPH radicals within a reasonable timeframe, resulting in no EC_50_ and N_DPPH_ value.

**Figure 7:**
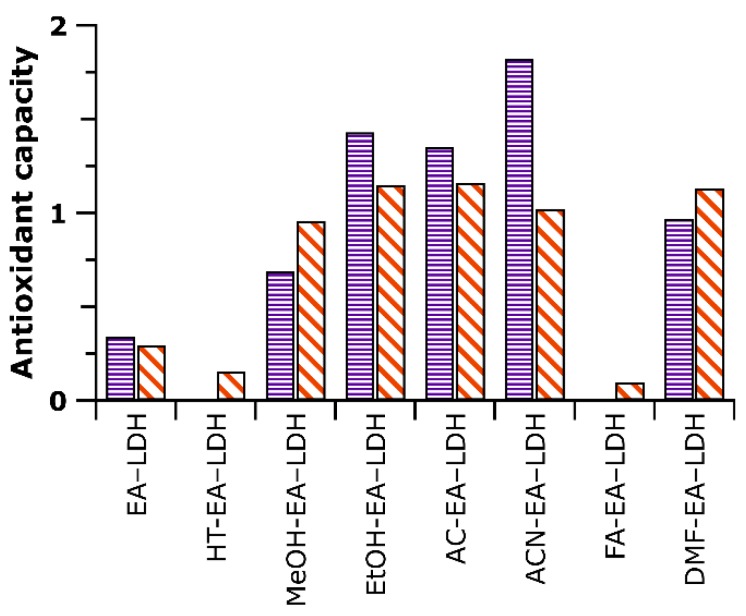
Antioxidant activity data determined in the DPPH and cupric reducing antioxidant capacity (CuPRAC) assays. The purple bars indicate N_DPPH_ values (with 3% error), while the orange striped ones belong to TEAC (with 4% error). Note that HT-EA-LDH and FA-EA-LDH did not decompose 50% of the DPPH radicals. Therefore, no N_DPPH_ values were calculated.

Scavenging efficacy can be quantified with the effective concentration (EC_50_) of EA, which correlates with an initial concentration of EA to react with half of the DPPH. This value can be given in a dimensionless form (proportionate to initial DPPH concentration) to estimate the number of reduced DPPH radicals by one antioxidant moiety (N_DPPH_, detailed in Equation S3) [45]. The entire data measured are displayed in Table 1 and Figure 7.

The values testify that even though the lactone form of EA was opened upon intercalation, the antioxidant activity was greatly preserved. Amongst LDH treated with AMOS, the ACN-EA-LDH and EtOH-EA-LDH showed the highest potential. Another remarkable result is that while EA had low solubility in water about 10 mg/L [60], the carrier LDH acted as a shield to form hydrophilic materials, applicable in much higher, nearly 100-fold (since 5 g/L EA-LDH suspensions may be used) doses, while still capable of effective DPPH scavenging. Note that the reaction between the saturated aqueous solution of EA and DPPH radicals was undetectable owing to the minimal change in the absorbance.

Although the N_DPPH_ values serve as a rough guide, one anionic EA moiety in the active EA-LDH hybrids typically react with 1–2 DPPH radicals via the intact phenolic –OH groups (Figure S6). The outstanding value for bare EA may be multifold. While the molecule only contains 4 –OH groups, nearly 12 molecules of DPPH is neutralized. This type of high activity of polyphenols is already known [45], the recombination of DPPH and a stabilized EA radical is possible.

The CuPRAC method [46] was also utilized to evaluate the antioxidant property of the materials in an aqueous medium. As detailed earlier, the Nc chelate of Cu(II) was formed in the cuvettes, and the antioxidant materials were added to reduce the central metal ions. The light blue complex turned into the orange-colored Cu(I)Nc_2_ upon reaction, whose scheme is shown in Figure S6. The antioxidant activity corresponds to the reducing capability of EA, i.e., the more potent materials generate more Cu(I) complex.

**Figure 8:**
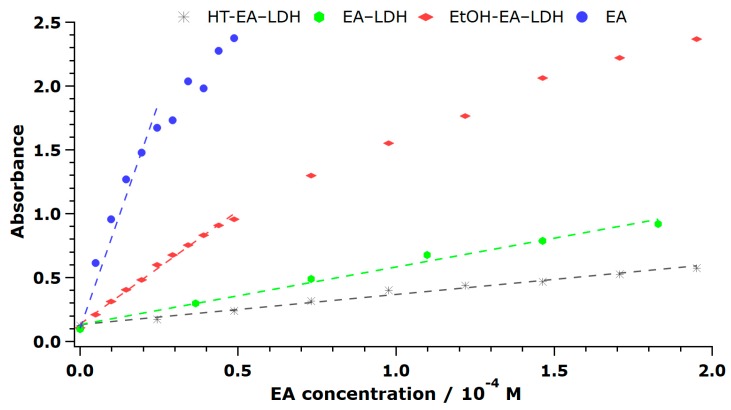
Formation of Cu(I)Nc_2_ complex, followed by the EA concentration dependence of the absorbance values. The measurements have an error of about 4%.

After registering the final absorbance of Cu(I)Nc_2_ after 30 min of reaction time using the bare and immobilized EA, it was concluded that the materials possessed different efficiency in the redox reactions, similar to the previously discussed DPPH test results. The unmodified LDH showed no activity (not shown). The final absorbances were plotted against the initial concentration of EA to unfold a linear correlation (Figure 8 and Figure S8), from which the slopes give the molar extinction coefficient (ε), given the path length was the same. Note that higher ε indicates higher antioxidant activity.

The tendencies in the data show that EA (as DMSO solution) had high activity, ACN-EA-LDH, MeOH-EA-LDH, EtOH-EA-LDH, AC-EA-LDH, and DMF-EA-LDH also showed good activity, while FA-EA-LDH and HT-EA-LDH had low activity in the assay indicating poor antioxidant features.

Generally, the obtained ε values are compared to the one reported for Trolox (a water-soluble analog of vitamin E, ε = 15400 M^–1^ cm^–1^ under the experimental conditions applied in the present study) [46]. Thus, Trolox served as a reference material within the CuPRAC assay (Figure S9). This value is called TEAC (Trolox equivalent antioxidant capacity), and the corresponding values are shown in Table 1 and Figure 7.

Similarly to the DPPH test, the solution of EA showed the best results with the TEAC value almost 5. Although intercalation decreased the reducing potential, the majority of AMOS-T EA-LDH was more active than the reference material Trolox. One plausible explanation is that the positively-charged Cu(II)Nc_2_ complex had a lower driving force to enter the interlamellar space due to electrostatic repulsion with the brucite-like layers. This repulsion might also lead to a reaction, in which the organic anions closer to the crystallite edges react more readily with the Cu(II) complex.

The potential desorption of the EA from the carrier LDH was assessed under test reaction conditions. It was concluded that typically 1–3 mass% of immobilized EA leaked from the LDH, with the exception of AC-EA–LDH, which released up to 6 mass% of its EA content (Table S4). The desorption was followed for 60 min, and the materials showed different characteristics, namely, continuous release (FA-EA-LDH, DMF-EA-LDH), sudden release (HT-EA-LDH, MeOH-EA-LDH, and EtOH-EA-LDH), and continuous release followed by re-adsorption (EA-EA-LDH, AC-EA-LDH, and ACN-EA-LDH). In accordance with the antioxidant capacity of bare EA, it was shown that the leaked EA contributed to the reduction in the antioxidant probes below 0.1%.

The BET surface areas of the LDH are collected in Table S3. The value of 73 m^2^ g^–1^ was typical for pristine LDH, while the effect of AMOS was striking. Accordingly, the samples with high activity also possessed a higher specific surface area (generally above 150 m^2^ g^–1^), resulting in better availability of the immobilized antioxidants. Washing with formamide and the hydrothermal treatment caused major collapse in the porous structure with < 10 m^2^ g^–1^ specific surface area, and hence, these LDH were the least active in the test reactions, i.e., they possessed poorer antioxidant activities in those cases. On the other hand, the direct correlation between EA release and surface area was not found. The changes in the surface properties of AMOS-LDH most likely occurred due to the partial exchange of interlamellar water molecules to organic ones leading to materials of different hydrogen bonding structures as well as with porosity alterations or partial exfoliation then restacking processes in certain solvents.

These above results clearly pointed out that the heterogenized EA kept its functional integrity. However, the extent of the scavenging ability depends on the structural features, such as porosity. The overall findings concluded from the structural and functional characterization of the samples revealed that the most effective materials are EtOH-EA-LDH, AC-EA-LDH, and ACN-EA-LDH. In general, the obtained composites are able to act as an efficient antioxidant in aqueous media, where the effectiveness of the free EA is impeded by its low solubility in water.

## 4. Conclusions

The coprecipitation method was successfully used as an intercalation route to incorporate polyphenolic EA into LDH containing Mg(II) and Al(III) metal ions. The structural features of the novel EA-LDH hybrid were tuned by subsequent washing with organic solvents. The intercalated ions were those of the opened forms of EA, as revealed in IR and UV-Vis measurements. The obtained hybrids kept their lamellar structures and contained a significant amount of EA irrespective of the treatment with organic solvents. The materials were capable of scavenging DPPH radicals and of reducing Cu(II)Nc_2_ complexes, indicating their high antioxidant activities. Upon intercalation, the modified LDH still acted as strong agents in the DPPH scavenging test reaction with 60 min reaction time to reach steady-state conditions. The active hybrids had an effective concentration, and the intercalated EA neutralized a significant amount of DPPH radicals. While in intercalated form, the reducing potential of EA decreased in CuPRAC assay, most of the modified LDH had higher activity than the typical reference molecule. Since EA is a phenolic compound with very poor water solubility, the hydrophilic LDH shell served as a protective environment to use this antioxidant without significant chemical modifications in aqueous media in higher concentrations. Therefore, the hybrids developed are promising candidates as in vitro and in vivo test materials against oxidative stress and in industrial manufacturing processes or product formulation, where the aim is to decrease the concentration of reactive oxygen species in aqueous medium.

## Figures and Tables

**Table 1:** Antioxidant activity values of the materials investigated.

Sample	EC_50_/10^–5^ M^a^	N_DPPH_^b^	ε/10^3^ M^–1^ cm^–1c^	TEAC^d^
EA	0.25	11.78	71.0	4.61
EA-LDH	8.73	0.34	4.5	0.29
HT-EA-LDH^e^	-	-	2.4	0.15
MeOH-EA-LDH	4.19	0.70	14.7	0.96
EtOH-EA-LDH	1.98	1.48	17.7	1.15
AC-EA-LDH	2.17	1.35	17.8	1.16
ACN-EA-LDH	1.61	1.82	15.7	1.02
FA-EA-LDH^e^	-	-	1.5	0.10
DMF-EA-LDH	3.01	0.97	17.4	1.13

^a^EA concentration needed to decompose 50% of the DPPH radicals. ^b^Number of DPPH decomposed by 1 EA. ^c^Molar extinction coefficient of the materials in the CuPRAC assay. ^d^Molar extinction coefficient of the materials compared to reference molecule Trolox in the CuPRAC assay. ^e^The composite was not able to decrease the DPPH concentration below 50% in a reasonable time frame.

## Supplementary Materials

The following are available online at https://www.mdpi.com/2076-3921/9/2/153/s1, **Figure S1**: XRD pattern of the LDH, and its composites prepared. The Miller indices are indicated, **Figure S2**: FT-IR spectra of LDH, its EA-intercalated form, and the hybrids modified with AMOS treatment, **Figure S3**: SEM micrograph of the LDH prepared: (**A**) LDH, (**B**) EA-LDH, (**C**) HT-EA-LDH, (**D**) MeOH-EA-LDH, (**E**) EtOH-EA-LDH, (**F**) AC-EA-LDH, (**G**) ACN-EA-LDH, (**H**) DMF-EA-LDH, (**I**) FA-EA-LDH. Scale bars represent 1 μm, **Figure S4**: UV-Vis spectra of EA at different protonated states, **Figure S5**: UV-Vis calibration curve of EA in 50 V% aqueous MeOH solution. The absorbance values were recorded at 350 nm, **Figure S6**: Reaction scheme between EA and DPPH (upper, purple border) and between EA and Cu(II)Nc_2_ (bottom, orange border) involving 2 –OH groups, **Figure S7**: Percentage of remaining (non-reacted) DPPH at steady-state as a function of the EA concentration applied. Data measured for all of the composites are shown. Measurements have an error of about 3%, **Figure S8**: The activity of the materials investigated in CuPRAC assay, as expressed in concentration-dependent absorbance values measured at 450 nm. Measurements have an error of about 4%, **Figure S9**: The reference activity of Trolox in CuPRAC assay expressed by the concentration-dependent absorbance values, **Table S1**: The position of the (003) diffraction peak and the corresponding basal spacing (d_0_) of the LDH prepared. The latter values were calculated with Equation S1. Hydrodynamic radii in water (R_h_, at 10 mg/L particle concentration) are also shown, **Table S2**: Characteristic IR bands of the solids investigated, **Table S3**: EA content and specific surface area of the organic-modified LDH, **Table S4**: Released amount of EA (immobilized mass%) from the obtained EA-LDH hybrids at different desorption periods.
