# Supplementary material for: Layered Double Hydroxide Nanoparticles to Overcome the Hydrophobicity of Ellagic Acid: An Antioxidant Hybrid Material
Source: Antioxidants (Basel). 2020 Feb 13;9(2):153. doi: 10.3390/antiox9020153 (PMC7070634; doi:10.3390/antiox9020153)
Supplement: Supplementary file 1 [file antioxidants-09-00153-s001.pdf]

Supplementary Materials

Layered Double Hydroxide Nanoparticles to Overcome the Hydrophobicity of Ellagic Acid: an Antioxidant Hybrid Material

Szabolcs Muráth[1,2], Adél Szerlauth[1], Dániel Sebők[3] and Istvan Szilagyi[1,2,*]

[1]MTA-SZTE Lendület Biocolloids Research Group, University of Szeged, H-6720 Szeged, Hungary; murathsz@chem.u-szeged.hu (S.M.); szerlauth.adel@gmail.com (A.S.); szistvan@chem.u-szeged.hu (I.S.)

[2]Interdisciplinary Excellence Center, Department of Physical Chemistry and Materials Science, University of Szeged, H-6720 Szeged, Hungary

[3]Interdisciplinary Excellence Center, Department of Applied and Environmental Chemistry, University of Szeged, H-6720 Szeged, Hungary; sebokd@chem.u-szeged.hu (D.S.)

*Correspondence: szistvan@chem.u-szeged.hu; Tel: +36 62 343255

Equations used for data evaluation

Bragg's law was used to calculate the basal spacing, i.e., the sum of the distance between two layers and the thickness of one layer in the LDH:

$$2d \cdot \sin\theta = n \cdot \lambda \tag{S1}$$

(d: basal spacing, θ: incidence angle of X-rays, n: positive integer, λ: wavelength of X-rays). For the (003), so-called first order reflection, n = 1.

The percentage of DPPH, which did not react in the samples, was provided by the Beer-Lambert correlation:

$$A = \varepsilon \cdot c \cdot \ell \text{ and } \mathrm{DPPH}\ (\%) = \frac{c_{final}}{c_{initial}} \cdot 100\% = \frac{A_{final}}{A_{initial}} \cdot 100\% \tag{S2}$$

(A: absorbance, ε: molar extinction coefficient, c: molar concentration of DPPH, ℓ: light path length).

The amount of DPPH scavenged by 1 mole of EA (N_{DPPH}) was calculated by the following correlations:

$$EC'_{50} = \frac{EC_{50}}{c_{EA,initial}} \qquad N_{DPPH} = \frac{1}{2 \cdot EC'_{50}} = \frac{1}{EC'_{100}} \tag{S3}$$

(EC'_{50}: dimensionless effective concentration of EA, EC_{50}: effective concentration of EA, $c_{EA,initial}$: the initial concentration of EA in the DPPH test, EC'_{100}: dimensionless value of the theoretical amount of EA needed to neutralize every DPPH radical).

Table S1. The position of (003) diffraction peak and the corresponding basal spacing (d_0) of the LDH prepared. The latter values were calculated with equation S1. Hydrodynamic radii in water (R_h, at 10 mg/L particle concentration) are also shown.

Sample	2θ (003)	d_0 (Å)	R_h (nm)
LDH	11.73	7.54	96
EA–LDH	11.40	7.76	286
HT-EA–LDH	11.41	7.75	474
MeOH-EA–LDH	11.41	7.75	325
EtOH-EA–LDH	11.25	7.86	282
AC-EA–LDH	11.57	7.65	299
ACN-EA–LDH	11.41	7.75	400
FA-EA–LDH	11.25	7.86	405
DMF-EA–LDH	11.41	7.75	559

Table S2. Characteristic IR bands of the solids investigated.

Wavenumber (cm^{-1})	Assignation	Sample
3500–3300	OH str	EA, LDH, EA–LDH
3200–3000	CH str<br>CC_{Ar} ovrt	EA, EA–LDH
1719	$C{=}O_{Lact}$ str	EA
1652	COO_{as} str	EA–LDH
1615, 1511	CC_{Ar} str	EA, EA–LDH
1400–1300	COO_{sy} str	EA–LDH
1357	EO str (in NO_3^-/CO_3^{2-})	LDH, EA–LDH
1350–1150<br>1100–1000	$C–O_{Lact, Ph}$ str	EA, EA–LDH
1120–1100	OH bnd	EA, EA–LDH
1100–1000	CH bnd<br>CC_{Ar}	EA, EA–LDH

str: stretching vibration, ovrt: overtones, as: asymmetric, sy: symmetric, bnd: bending vibration, Ar: aromatic, Lact: lactone bond, Ph: phenolic. Note: the presence of NO_3^- may arise from precursors, while CO_3^{2-} from airborne surface adsorption.

Table S3. EA content and specific surface area of the organic-modified LDH.

Sample	EA content (mass %)	BET specific surface area (m^2g^{-1})
EA–LDH	17.1	<10
HT-EA–LDH	18.9	<10
MeOH-EA–LDH	17.2	176
EtOH-EA–LDH	16.6	239
AC-EA–LDH	17.2	79
ACN-EA–LDH	16.4	136
FA-EA–LDH	14.7	<10
DMF-EA–LDH	16.9	223

Note: the specific surface area of pristine LDH was 73 m^2g^{-1}.

Table S4. Released amount of EA (immobilized mass%) from the obtained EA–LDH hybrids at different desorption periods.

Sample	5 mins	10 mins	20 mins	40 mins	60 mins
EA–LDH	0.7	1.5	1.9	0.9	0.9
HT-EA–LDH	1.1	0.9	1.0	1.1	1.0
MeOH-EA–LDH	2.3	2.4	2.4	2.4	2.4
EtOH-EA–LDH	2.4	2.4	2.4	2.7	2.4
AC-EA–LDH	5.9	6.0	5.4	3.7	2.5
ACN-EA–LDH	3.3	3.4	3.4	3.3	2.8
FA-EA–LDH	2.2	2.2	2.4	2.7	2.8
DMF-EA–LDH	2.1	2.3	2.4	2.7	2.9

Note: the leaked EA had negligible contribution to the measured antioxidant efficiencies.

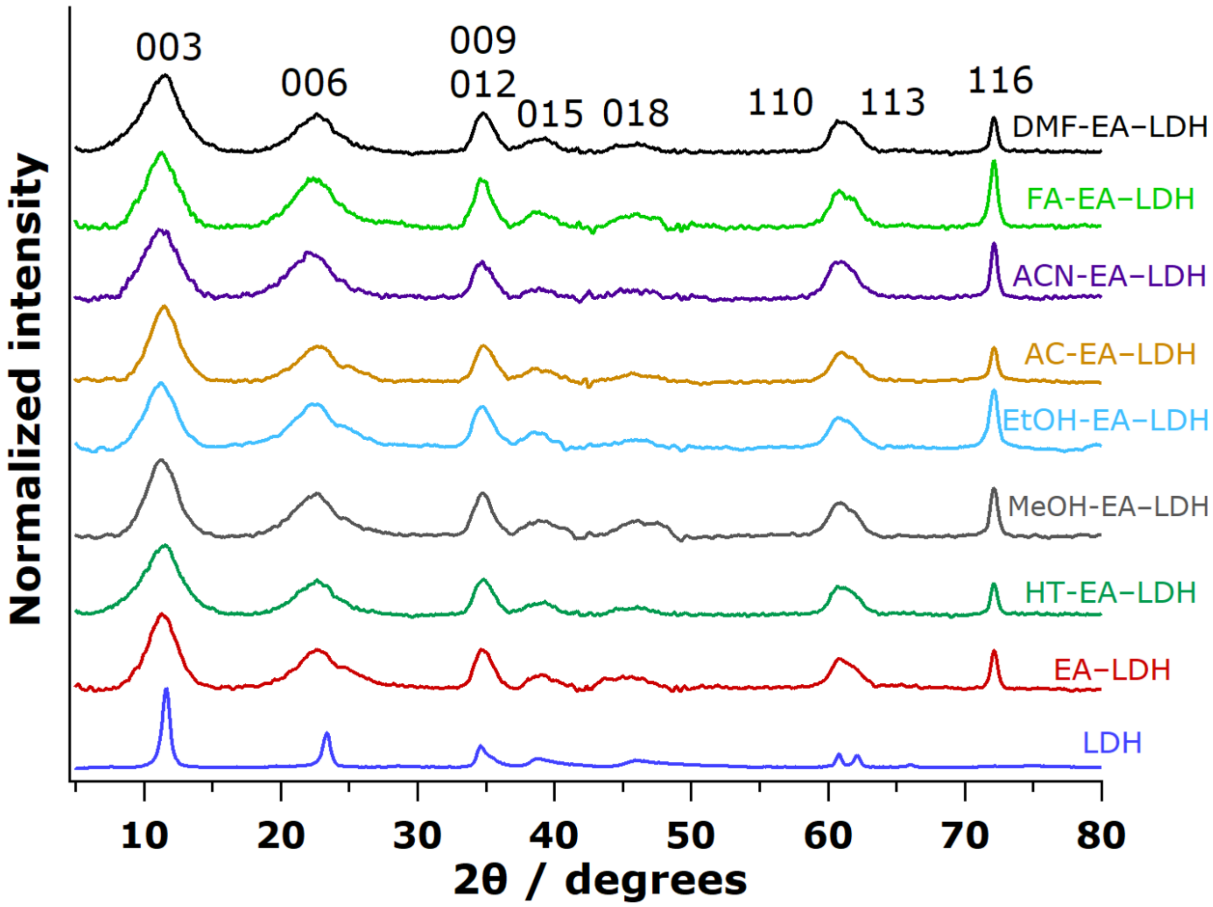

Figure S1. XRD pattern of the LDH and its composites prepared. The Miller indices are indicated.

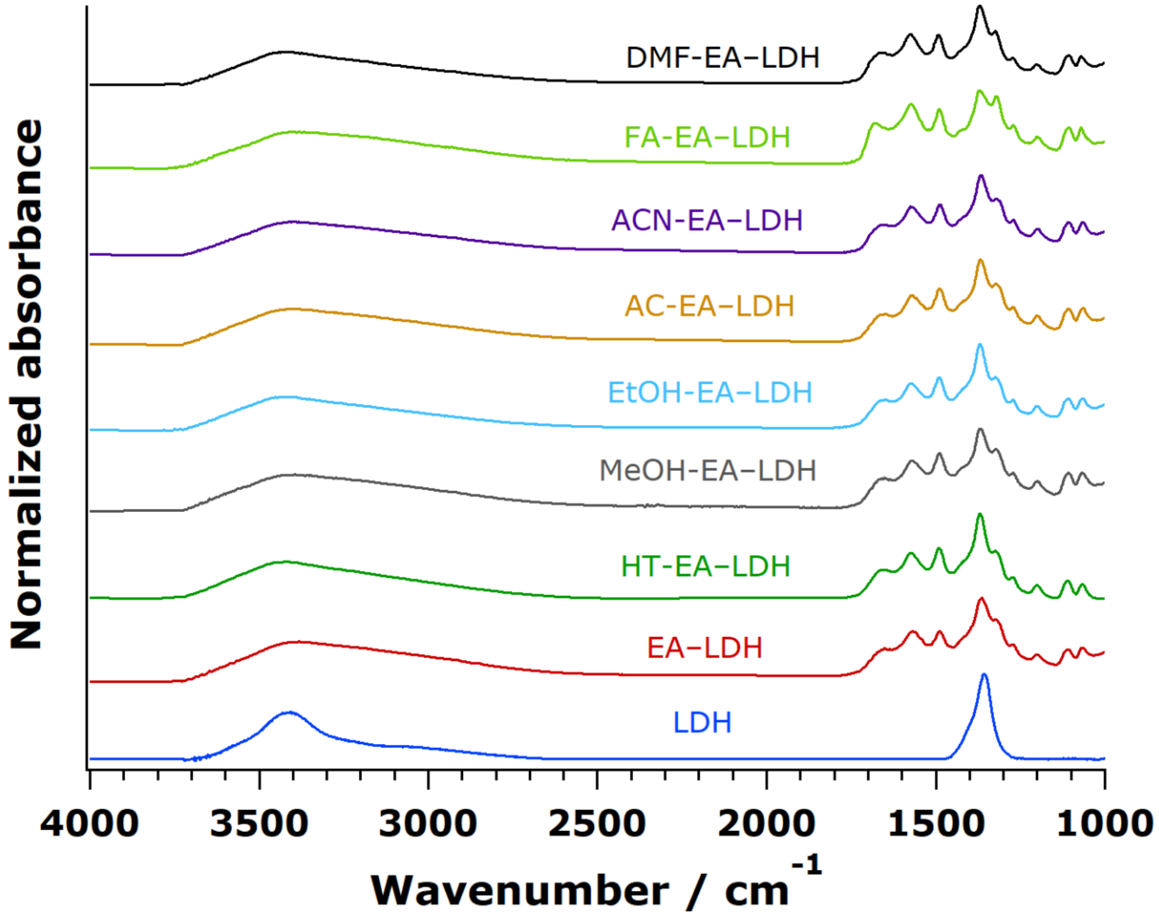

Figure S2. FT-IR spectra of the LDH, its EA-intercalated form and the hybrids modified with AMOS treatment.

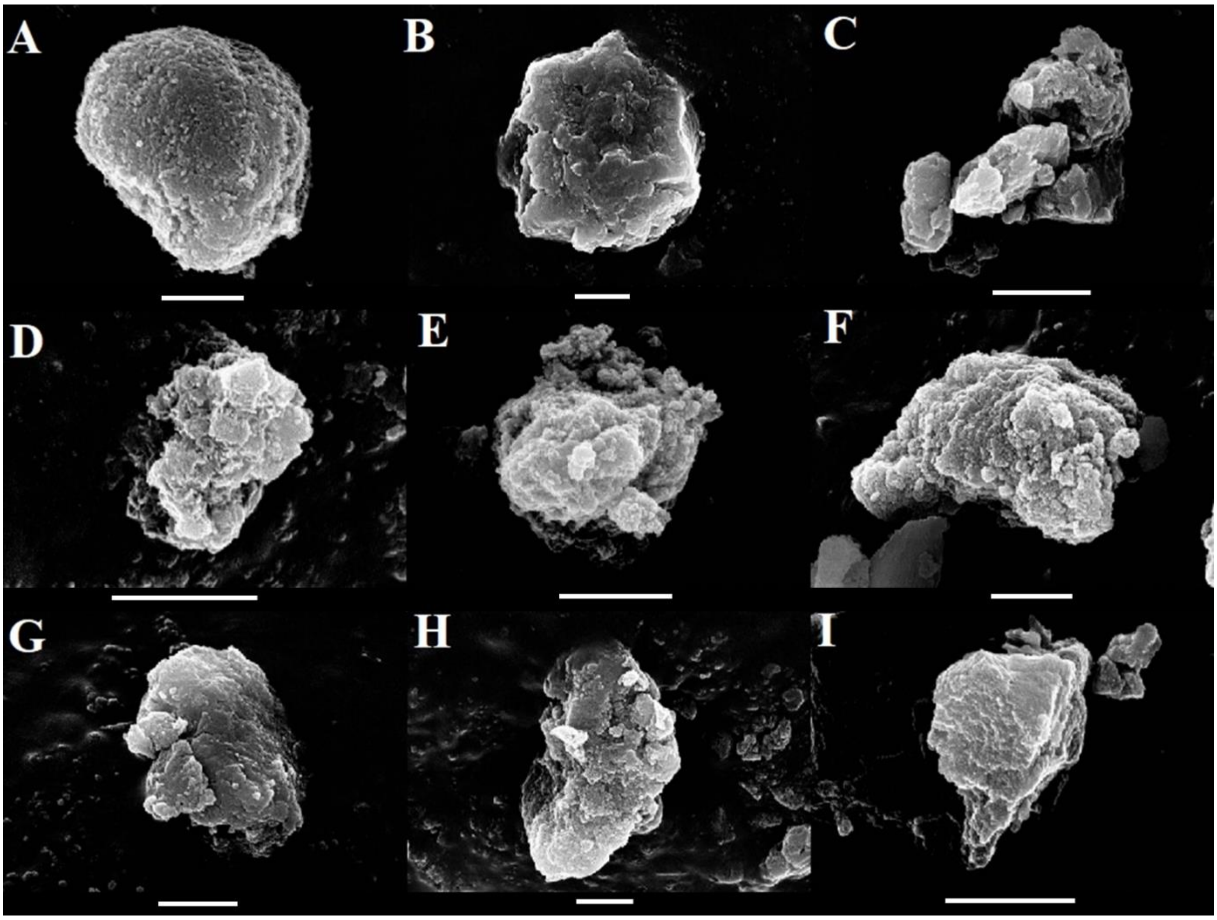

Figure S3. SEM micrograph of the LDH prepared: (A) LDH, (B) EA–LDH, (C) HT-EA–LDH, (D) MeOH-EA–LDH, (E) EtOH-EA–LDH, (F) AC-EA–LDH, (G) ACN-EA–LDH, (H) DMF-EA–LDH, (I) FA-EA–LDH. Scale bars represent 1 μm.

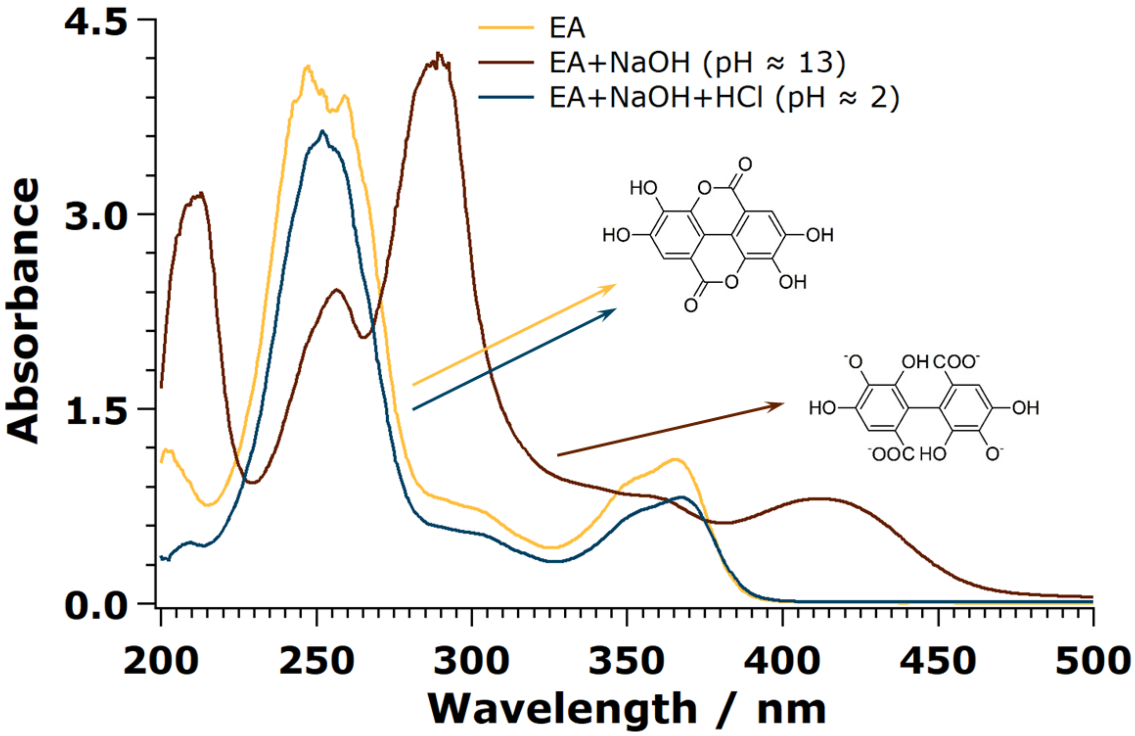

Figure S4. UV-Vis spectra of EA at different protonated states.

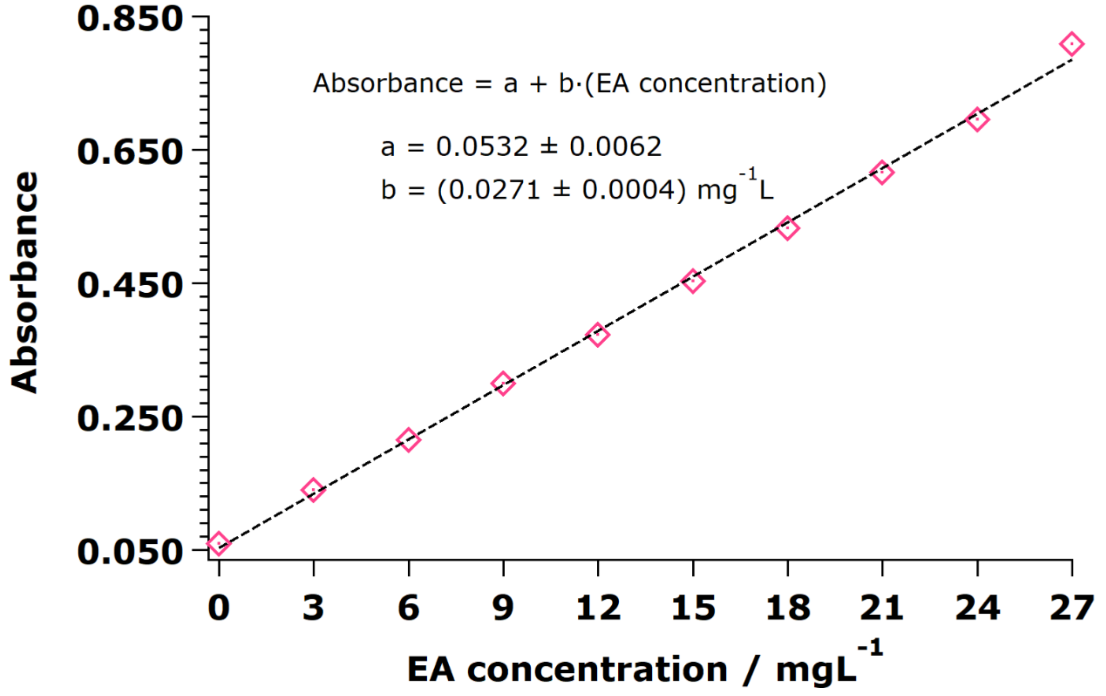

Figure S5. UV-Vis calibration curve of EA in 50 V% aqueous MeOH solution. The absorbance values were recorded at 350 nm.

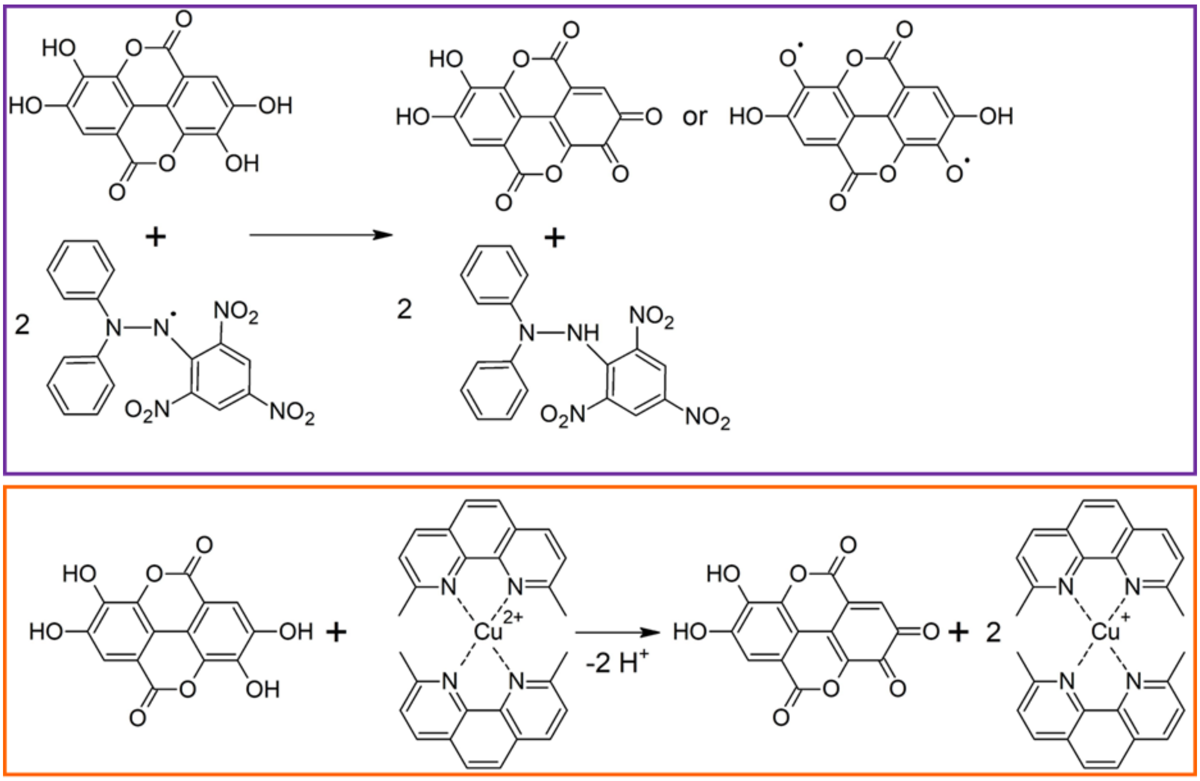

Figure S6. Reaction scheme between EA and DPPH (upper, purple border) and between EA and Cu(II)Nc$_2$ (bottom, orange border) involving 2 –OH groups

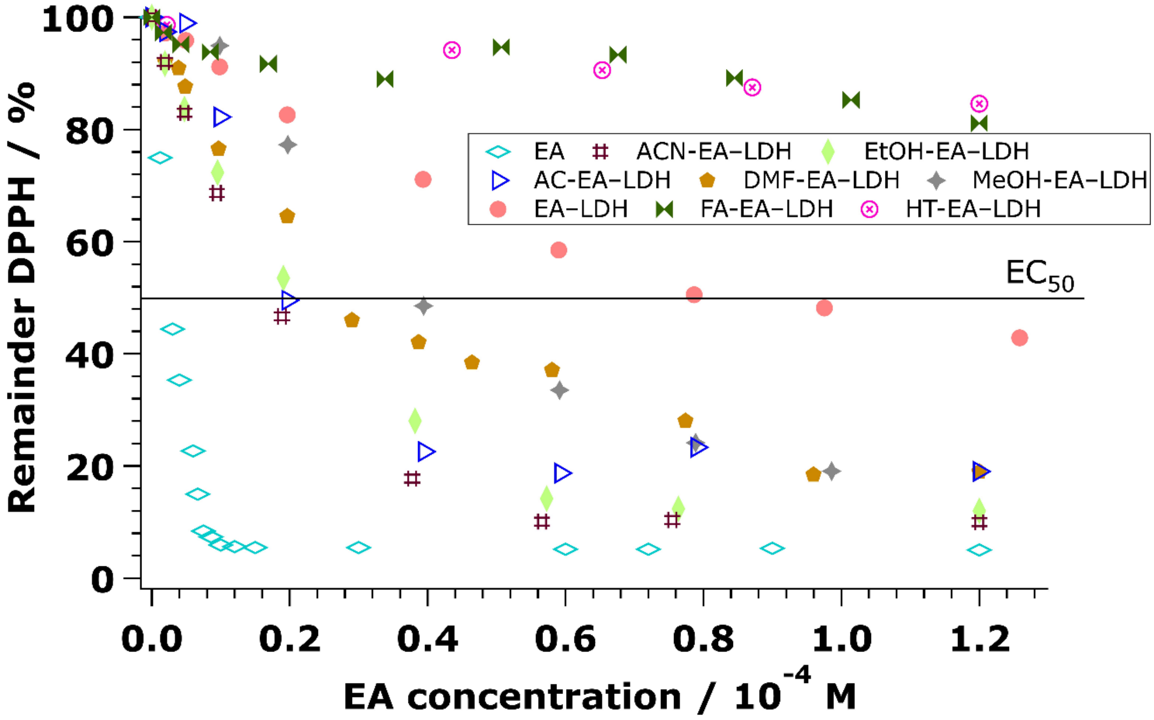

Figure S7. Percentage of remaining (non-reacted) DPPH at steady-state as a function of the EA concentration applied. Data measured for all of the composites are shown. Measurements have an error of about 3%.

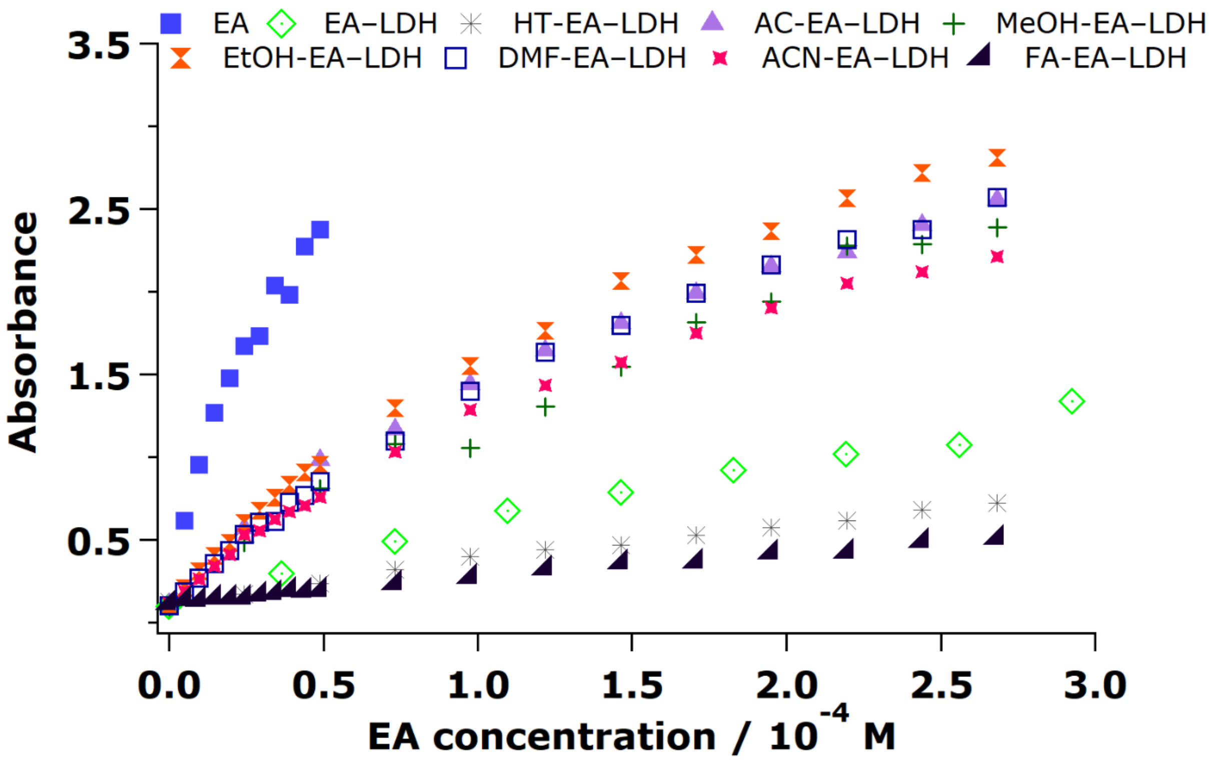

Figure S8. The activity of the materials investigated in CuPRAC assay, as expressed in concentration dependent absorbance values measured at 450 nm. Measurements have an error of about 4%.

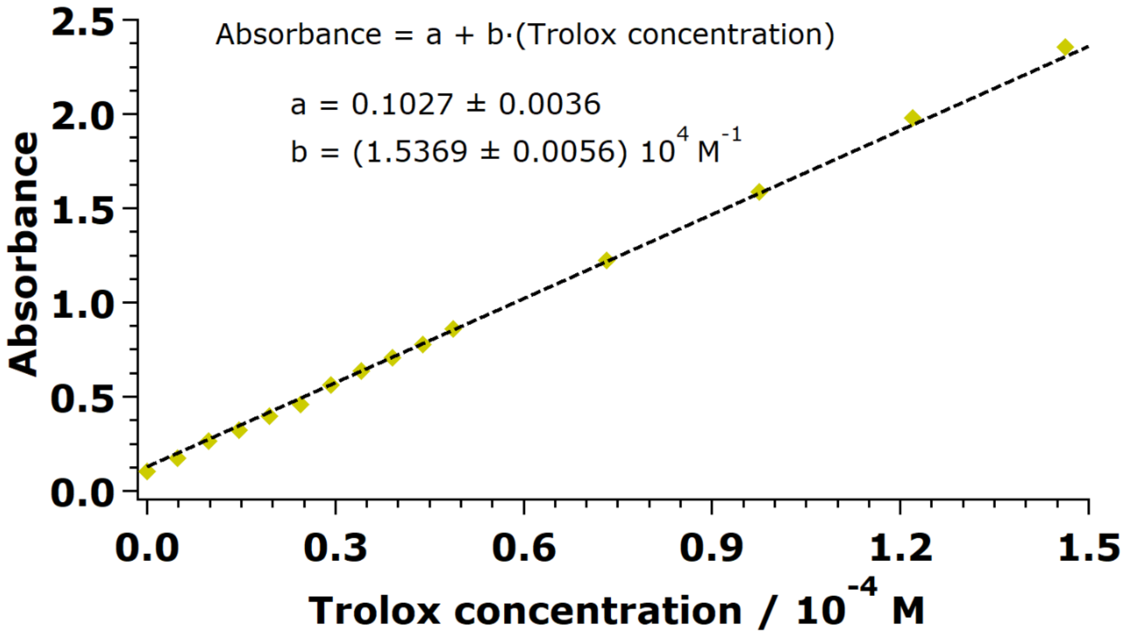

Figure S9. The reference activity of Trolox in CuPRAC assay expressed by the concentration dependent absorbance values.
